# Nuclear Receptor Coactivators (NCOAs) and Corepressors (NCORs) in the Brain

**DOI:** 10.1210/endocr/bqaa083

**Published:** 2020-05-25

**Authors:** Zheng Sun, Yong Xu

**Affiliations:** 1 Department of Molecular and Cellular Biology; Baylor College of Medicine, Houston, Texas; 2 Department of Medicine, Division of Diabetes, Endocrinology and Metabolism; Baylor College of Medicine, Houston, Texas; 3 USDA/ARS Children’s Nutrition Research Center, Department of Pediatrics; Baylor College of Medicine, Houston, Texas

**Keywords:** steroid receptor coactivator (SRC), nuclear receptor corepressor (NCOR), neuroendocrine, behavior, brain

## Abstract

Nuclear receptor coactivators (NCOAs) and corepressors (NCORs) bind to nuclear hormone receptors in a ligand-dependent manner and mediate the transcriptional activation or repression of the downstream target genes in response to hormones, metabolites, xenobiotics, and drugs. NCOAs and NCORs are widely expressed in the mammalian brain. Studies using genetic animal models started to reveal pivotal roles of NCOAs/NCORs in the brain in regulating hormonal signaling, sexual behaviors, consummatory behaviors, exploratory and locomotor behaviors, moods, learning, and memory. Genetic variants of NCOAs or NCORs have begun to emerge from human patients with obesity, hormonal disruption, intellectual disability, or autism spectrum disorders. Here we review recent studies that shed light on the function of NCOAs and NCORs in the central nervous system.

Nuclear receptors (NRs) are a family of transcription factors with more than 50 members in the human genome (1, 2). Unlike most other transcription factors, NRs can be directly bound and modulated by small-molecule ligands, which makes NRs druggable transcription factors. The ligands for NRs are diverse, including endogenous hormones, metabolites, drugs, and xenobiotics. These ligands can work as agonists, antagonists, or inverse agonists in regulating the downstream gene transcription. Nuclear receptor coregulators can bind to NRs in a ligand-dependent manner, thus recruiting epigenome-modifying enzymes or chromatin remodelers (3). These molecular events ultimately lead to assembly or expelling of the RNA polymerase-containing transcriptional machinery for initiating or terminating gene transcription. The relative abundance and functional interplay between NRs and their coregulators determine the net transcriptional outcome of cellular responses to ligands.

Nuclear receptor coregulators can be categorized into nuclear receptor coactivators (NCOAs) and corepressors (NCORs), depending on the originally identified ability to activate or repress gene transcription, respectively (3). However, these names do not accurately reflect their functions as accumulating evidence suggests that the direction of transcriptional regulation is not an intrinsic feature of the coregulators but rather highly context-dependent (4-6). NCOAs and NCORs have been extensively studied in the fields of endocrinology, metabolism, cancer, and development (7-9). Their functions in the central nervous system (CNS) in adult mammals are relatively less well characterized (10, 11). Here we review the studies exploring the functions of NCOAs and NCORs in the brain with the emphasis on the recent *in vivo* studies in mammals.

## Molecular Architecture

### The NCOAs complex

**Figure 1.**
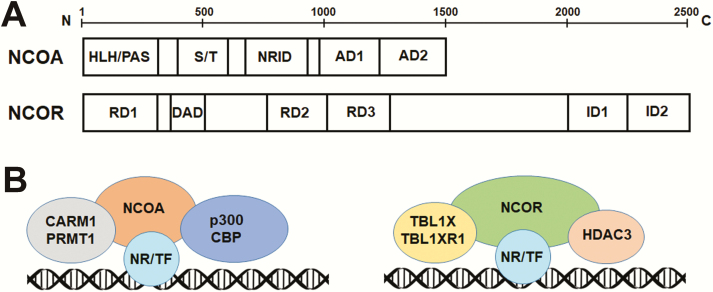
**Molecular architecture of NCOAs and NCORs. (A)** NCOAs are composed of the basic helix–loop–helix-PER-ARNT-SIM (HLH/PAS) domain on the N-terminus, a serine/threonine-rich (S/T) domain and a nuclear receptor interaction domain (NRID) in the middle, and 2 transactivation domains (AD) on the C-terminus. NCORs have 3 repression domains (RD1, RD2, and RD3), a deacetylase activation domain (DAD), and 2 interaction domains (ID1 and ID2) that interact with nuclear receptors. **(B)** Nuclear receptors (NRs) or other transcription factors (TFs) recruit NCOAs or NCORs to the genome, which further recruit epigenome modifiers and readers, chromatin remodelers, and the transcription initiation machinery.

NCOA1, NCOA2, and NCOA3 belong to the p160 steroid receptor coactivator (SRC) family with similar amino acid sequences and domain architecture (7). NCOA1 is also known as SRC-1 or receptor interacting protein 160 (RIP160), or lysine acetylatrase 13A (KAT13A). NCOA2 is also known as SRC-2, glucocorticoid receptor-interacting protein 1 (GRIP1), or transcriptional mediators/intermediary factor 2 (TIF2). NCOA3 is also known as SRC-3, amplified in breast 1 (AIB1), ACTR, thyroid hormone receptor activator molecule 1 (TRAM-1), receptor-associated coactivator (RAC3), or CBP-interacting protein (pCIP). NCOA1 through 3 are composed of the basic helix–loop–helix-PER-ARNT-SIM (HLH-PAS) domain on N-terminus, a serine/threonine-rich (S/T) domain and a nuclear receptor interaction domain (NRID) in the middle, and 2 transactivation domains (AD) on the C-terminus (12, 13) (Fig. 1A). The HLH-PAS domain contains the nuclear localization signal. The HLH-PAS is homologous to DNA-binding regions of the other HLH-containing transcription factors, but there is currently no report about NCOA1-3 directly binding to DNA. Instead, the HLH-PAS domain can bind to transcription factors, other coactivators, or SWI/SNF chromatin remodelers (14). The S/T domain is enriched with sites for phosphorylation by a variety of different kinase cascades, including PKA, Src, MAPK, ERK, GSK3β, and CK (12, 15). The NRID contains 3 LXXLL motifs (where L is leucine, X is any amino acid) that are responsible for interaction with nuclear receptors such as thyroid hormone receptor (TR), estrogen receptor (ER), androgen receptor (AR), progesterone receptor (PR), glucocorticoid receptor (GR), and retinoid X receptor (RXR) (12, 15). Many nuclear receptors can interact with other regions of NCOAs in addition to the NRID, and the NRID can also bind to other transcription factors in addition to nuclear receptors. The AD1 can interact with histone acetyltransferases such as CBP and p300. The AD2 can interact with coactivator-associated arginine methyltransferase 1 (CARM1) and protein arginine methyltransferase 1 (PRMT1) (12, 15) (Fig. 1B). NCOAs are subjected to a variety of posttranslational modifications including phosphorylation, acetylation, methylation, ubiquitination, and SUMOylation throughout different domains and regions (12, 16). NCOA4, 5, and 6 are distantly related to NCOA1-3, and their functions in the CNS are unknown. We focus on NCOA1-3 in this review.

### The NCORs complex

NCOR1 and NCOR2 share similar sequences and domain architecture. NCOR2 is also known as silencing mediator for retinoid and thyroid hormone receptor (SMRT). NCORs have 3 repression domains (RD1, RD2, and RD3) at the N-terminus and two interaction domains (ID1 and ID2) at the C terminus (17) (Fig. 1A). RDs can interact with epigenome-modifying enzymes and chromatin remodelers, while IDs can interact with different nuclear receptors (17).

Histone deacetylase 3 (HDAC3), a class I HDAC, is the major HDAC that confers the deacetylase enzyme activity to the NCOR complex. The enzyme activity of HDAC3 is dependent on binding to the deacetylase activating domain (DAD) on the N-terminus of NCOR1/2(18) (Fig. 1A**).** Such binding causes a conformational change in HDAC3 protein, which is essential for its deacetylase catalytic activity (19). Purified HDAC3 protein shows minimal enzyme activity in the absence of DAD (20). In addition to DAD, a region near the C-terminus of NCOR1/2 can also interact with HDAC3 without affecting HDAC3 enzymatic activity (21). Class IIa HDACs (HDAC4, HDAC5, HDAC7, and HDAC9) are also associated with the NCOR complex, but they have no intrinsic deacetylase enzymatic activity. As a result, their enzymatic activities are dependent on HDAC3 (22). HDAC3 is exclusively found in the NCOR complex and is considered as a stable and essential component of the NCOR complex.

Transducin β-like 1 X-linked/transducin β-like 1 X-linked receptor 1 (TBL1X/TBL1XR1) is another stable component of the NCOR complex and is essential for the transcriptional function of NCORs. TBL1XR1 has about 86% identity with TBL1X based on the amino acid sequence. Both TBL1X and TBL1XR1 have 6 WD-40 domains and are stable components of the NCOR complex based on protein co-IP experiments (23, 24). They interact with NCORs through the WD-40 repeats, as well as the N-terminal region (23, 24) (Fig. 1B). Conversely, NCORs interact with TBL1X/TBL1XR1 through the RD1 region and a region between RD3 and ID1(23, 24). TBL1XR1 selectively mediates the exchange of NCOR for NCOA upon NRs binding to the ligand, which is required for ligand-induced transcriptional activation (25). In addition to the NCOR/NCOA switch, TBL1X/TBL1XR1 also targets NCORs for ubiquitination and degradation after NR ligand binding (25). Therefore, TBL1X/TBLXR1 regulates the chromatin occupancy of NCORs both positively and negatively, depending on the presence of the ligand for the NRs that recruit the NCOR complex.

### Dynamics of NCOAs and NCORs

The protein-DNA complexes of NR and coregulators are dynamic. For example, unliganded ER binds to NCORs, while 17β-estradiol promotes the formation of a chemically stable complex in a “poised” status composed of NCOA and some atypical corepressors. Further phosphorylation on NCOAs by DNA-dependent protein kinase or other NCOA modifications activate the complex by recruiting epigenome modifiers such as p300 and CARM1, which further recruits epigenome readers or remodelers (12, 26, 27). Most epigenome modifiers, readers, and remodelers are flexible and can participate in a variety of different coregulator complexes (28). Therefore, they are not discussed in detail in this review. In contrast, the evidence so far suggests that HDAC3 and TBL1X/TBL1XR1 work exclusively in the NCOR complex; therefore, they are included in this review.

## Expression in the Mammalian Brain

NCOAs and NCORs are widely expressed in adult mammalian brains. In situ hybridization in adult mouse brains showed that NCOA1 is highly expressed in the olfactory bulb, hippocampus (CA1, CA2, and CA3 regions and dentate gyrus), piriform cortex, amygdala, hypothalamus, cerebellum (in the Purkinje cell layer but not in the granule and molecule layers), and brainstem (especially facial nucleus, trigeminal nucleus, and hypogrossal nucleus) (29). NCOA1 is also expressed in the thalamus and neocortex. In most brain structures where NCOA1 is expressed, NCOA2 is also expressed, albeit at lower levels. In particular, NCOA2 expression is high in the anterior pituitary (29). In the hypothalamus, NCOA2 mRNA is expressed in the medial preoptic area (MPOA), ventromedial nucleus (VMH), arcuate nucleus (ARC), bed nucleus of the stria terminalis, supraoptic nucleus, and suprachiasmatic nucleus (30). NCOA3 mRNA is detectable only in the hippocampus. There may be compensatory regulation of the expression levels among NCOAs. For example, NCOA2 mRNA is found slightly elevated in the cerebellum Purkinje cells in the NCOA1^-/-^ mice (31).

NCOA1 has at least 2 different splicing isoforms, designated 1a and 1e. Although both 1a and 1e mRNA are expressed in many of the abovementioned brain areas, including the hippocampus, amygdala, hypothalamus, basal ganglia, and neocortex, there are differences in several regions. NCOA1a mRNA are found at higher levels in the anterior pituitary, ARC, paraventricular nucleus (PVN), VMH, locus coeruleus, and the trigeminal motor nucleus, which are all important targets of steroid hormones. In comparison, NCOA1e mRNA levels are slightly higher in the caudal nucleus accumbens, basolateral amygdala, and some thalamic nuclei (29).

NCOAs gene expression is modulated by developmental processes, aging, hormonal signals, and the environment (10). There are time-, region-, and gene-specific regulations of NCOAs expression during postnatal development in the hippocampus (32). Hippocampal NCOA1 is downregulated by orchiectomy in male mice, and such downregulation can be rescued by treatment with testosterone (33). Aging or ER antagonists were shown to downregulate NCOA1 in the hippocampus of female mice, while 17β-estradiol treatment can upregulate NCOA1. Treatment with 17β-estradiol in female mice also increased NCOA1 protein levels in the ARC, but not the MPOA or the VMH. By comparison, 17β-estradiol does not alter NCOA2 protein levels in these brain regions (34). Corticosterone exposure downregulates NCOA1 gene expression in the PVN and central amygdala (CeA), and upregulates NCOA1 gene expression in the ventral hippocampus in rats that show posttraumatic stress avoidance behaviors (35).

NCOR1 and NCOR2 are widely expressed in the adult mouse brain with overall similar neuroanatomical distribution (36). Immunohistochemistry detected both proteins in all cortical layers of the neocortex, the pyramidal cell layer of the piriform cortex, all subregions of the hippocampus, and striatum. Both NCOR1 and NCOR2 are expressed in glutamatergic and GABAergic neurons with no preferential expression in a specific neuronal population versus the other. Microglial and oligodendrocytes in the cortex, hippocampus, and striatum show detectable expression of NCOR1 but not NCOR2 (36).

## Genetic Mammalian Models

### Sexual behaviors

Brain NCOAs are involved in 17β-estradiol and progesterone-induced sexual behaviors. Androgen treatment in neonatal female rats can increase male-typical behaviors such as mount or intromission, and reduce female-typical behaviors such as lordosis. Neonatal injection of NCOA1 antisense oligonucleotides (ASO) in rats blunts defeminizing actions of androgen in female rats and increases lordosis in male rats (37). Infusing 17β-estradiol and/or progesterone induces sexual behaviors in female ovariectomized (OVX) rats. Acute knockdown of NCOA1 or NCOA2, but not NCOA3, in the VMH through ASO injection in female rats reduces the estradiol/progesterone-induced lordosis behaviors (38) as well as progesterone-induced ear wiggling, hopping, and darting behaviors (39). Studies in quail also suggest that knockdown of NCOA1 in the preoptic area and hypothalamus blunts steroid-dependent male-typical sexual behaviors and the associated neuroplasticity (40).

### Energy homeostasis

Other than sexual behaviors, 17β-estradiol also regulates food intake and energy homeostasis. Administration of 17β-estradiol reduces food intake and body weight in OVX female mice. These effects of 17β-estradiol on energy homeostasis are significantly blunted in female NCOA1 whole-body knockout (NCOA1^-/-^) mice (41). These results suggest that NCOA1 is required for the antiobesogenic effects of 17β-estradiol. Mechanistically, NCOA1 interacts with transcription factor STAT3, leading to enhanced Proopiomelanocortin (POMC) gene transcription in the hypothalamus. STAT3 and POMC are known downstream effectors of the anorexic hormone leptin. The deletion of NCOA1 in POMC neurons attenuates their depolarization by leptin, decreases POMC expression, and increases food intake, which accelerates high-fat diet-induced obesity. Knock-in mice carrying a loss-of-function variant (NCOA1^L1376P^) identified in human obese patients show lower POMC gene expression, impaired leptin-induced depolarization of POMC neurons, increased food intake, and increased susceptibility to obesity (42). In line with the role of NCOA1 in regulating STAT3 in the brain, the interplay between NCOA1 and STAT family of transcription factors has also been characterized in the context of carcinogenesis (43-47). Consistent with the finding in mice, NCOA1 gene expression at the nucleus of the solitary tract (NTS) is decreased by high-fat diet in OVX rats (48). Knockdown of NCOA1 using lentivirus small hairpin RNA (shRNA) injected at the NTS in OVX rats does not significantly change body weight in the absence of 17β-estradiol, but increased food intake and body weight in the presence of 17β-estradiol administration. These results suggest that NCOA1 in the NTS also contributes to the anorectic action of 17β-estradiol (48).

In addition to NCOA1, NCOA2 and NCOA3 also play nonredundant functions in the CNS. One interesting phenotype of NCOA2^-/-^ mice is disrupted diurnal rhythm in locomotor activity and food intake behaviors. NCOA2^-/-^ mice exhibit a bimodal wheel-running pattern on normal 12h light/12h dark (LD) condition, with abnormally elevated physical activity occurring late in the light phase (49). As a result, the running activity of NCOA2^-/-^ mice is scattered, with 40% in the light phase and only 60% in the dark phase, compared to wild-type (WT) mice with over 90% of activity in the dark phase. Similarly, food intake also show a phase advance in NCOA2^-/-^ mice (49).

### Mood regulation

In addition to disrupted energy homeostasis, NCOA1^-/-^ mice show reduced anxiety-like behaviors in males but not in females, compared with WT littermates (50) (Table 1). Glucocorticoid signaling can regulate stress response, anxiety, and fear. GR upregulates corticotropin-releasing hormone (CRH) expression in the central amygdala (CeA), which modulates anxiety-like behavior and memory (51). As a negative feedback loop, GR also suppresses CRH expression in the hypothalamic PVN, which further suppresses adrenocorticotropic hormone (ACTH) production from the anterior pituitary gland. NCOA1^-/-^ mice have a normal acute rise in corticosterone after ACTH administration, but show elevated circulating corticosterone despite normal ACTH after exposure to stress, suggesting increased adrenal sensitivity to stress and a concomitant defect in glucocorticoid-mediated feedback inhibition (52). Another study found that NCOA1^-/-^ mice display lower basal CRH mRNA levels in the CeA and upregulated hypothalamic CRH after chronic stress (53). Therefore, NCOA1 is involved in both gene upregulation and downregulation in response to glucocorticoids.

**Table 1.** Genetic Mammalian Models of the NCOAs and NCORs

Genetic Manipulation	Phenotype	Ref.
NCOA1 knockdown	NCOA1 ASO blunted defeminizing actions of androgen in female rats and increased lordosis in male rats.	(37)
	NCOA1 or NCOA2 knockdown of in the VMH in adult female rats reduced the estradiol/progesterone-induced lordosis behaviors.	(38)
	NCOA1 knockdown in the VMH reduced progesterone-induced ear wiggling, hopping, and darting behaviors in female rats.	(39)
	NCOA1 knockdown using lentivirus small hairpin RNA in the nucleus of the solitary tract in OVX female rats increased food intake and body weight in the presence of 17β-estradiol administration.	(48)
	NCOA1 knockdown in the adult mouse hippocampus through injection of the lentiviral shRNA impaired spatial learning and memory, decreased the CA1 synapse density, and impaired long-term potentiation.	(69)
NCOA1 splicing isoform switch	Injection of “exon-skipping” ASO targeting on in the CeA increased NCOA1a/1e ratio, blunted glucocorticoid-induced exploratory behavior, and attenuated context-dependent fear memory.	(55, 56)
NCOA1 global knockout	NCOA1^-/-^ mice show blunted effects of 17β-estradiol in reducing food intake and body weight in OVX female mice.	(41)
	NCOA1^-/-^ mice show elevated circulating corticosterone despite normal ACTH after exposure to stress, suggesting defective glucocorticoid-mediated feedback inhibition in the CNS.	(52)
	NCOA1^-/-^ mice display lower basal CRH gene expression in the CeA and upregulated hypothalamic CRH expression after chronic stress.	(53)
	NCOA1^-/-^ mice display elevated circulating levels of TSH, T4, and T3, and required higher doses of T3 to suppress TSH and T4, suggesting reduced sensitivity of the thyrotrophs in the anterior pituitary to TH.	(63, 64)
	NCOA1^-/-^ mice show reduced anxiety, impaired motor learning, and lower pain threshold in males.	(31, 50)
NCOA1^L1376P^ knock-in	NCOA1^L1376P^ knock-in mice bearing a mutation identified in obese patients show decreased leptin-induced depolarization of POMC neurons, increased food intake, and exacerbated obesity.	(42)
NCOA2 global knockout	NCOA2^-/-^ mice show lower body weight, lower anxiety, impaired motor learning and motor coordination, and higher pain threshold in females. NCOA2^-/-^ male mice show deficits in sensorimotor gating.	(50)
	NCOA2^-/-^ mice show disrupted diurnal rhythm in locomotor activity and food intake behaviors, with abnormally elevated physical activity occurring late in the light phase.	(49)
NCOA3 global knockout	NCOA3^-/-^ mice show reduced amino acid levels in the brain, with increased anxiety behaviors in females, and higher pain threshold in both sexes.	(50,13)
NCOR1 knockdown	NCOR1 knockdown in the amygdala by siRNA in rats increases juvenile social play behaviors in males and anxiety-like behaviors in both males and females. Social interaction in the three-chamber assay is normal in these rats.	(60)
NCOR1 truncation	Global deletion of the last few exons of NCOR1 (NCOR1ΔID) in mice leads to lower circulating TH levels without a compensatory increase in TSH production. Pituitary-specific expression of NCOR1ΔID shows a similar phenotype.	(66,67)
NCOR1^Y478A^ NCOR2^Y470A^ (NS-DADm)	NS-DADm mice show increased activity, enhanced locomotor coordination, reduced anxiety, impaired social interaction, and impaired spatial learning and recognition memory.	(70)
Vgat-Cre; NCOR1^loxP^; NCOR2^loxP^	NS-V mice show impaired learning and memory due to hyperexcitability of the lateral hypothalamus to hippocampal CA3 neural projection.	(70)
Virus-mediated region-specific deletion of HDAC3	HDAC3 depletion in the adult hippocampal CA1 region enhances long-term memory formation (71).	(71)
	HDAC3 depletion in the adult lateral hypothalamus impairs memory, while HDAC3 depletion in the adult hippocampal CA3 region does not affect memory.	(70)
	HDAC3 depletion in the nucleus accumbens enhances cocaine-induced conditioned place preference (CPP) acquisition.	(72)
	HDAC3 depletion in retinal ganglion cells (RGC) in adult mice ameliorates cell death, heterochromatin formation, nuclear envelope breakdown, and nuclear pore damage in RGCs following optic nerve crush.	(73)
Nestin-Cre, HDAC3^loxP^	These mice show abnormal cytoarchitecture of the neocortex and cerebellum that leads to lethality within a day after birth.	(75)
Camk2a-Cre, HDAC3^loxP^	These mice show neuron loss in the hippocampus, hyperactivity, abnormal exploratory behavior, hind limb clasping, sociability deficits, and impaired memory, with reduced FOXO activity. A separate study reports progressive hindlimb paralysis, ataxia, higher numbers of astrocytes, and Purkinje neuron degeneration, which leads to lethality at ~ 6 weeks old.	(75, 76)
Emx1-Cre, HDAC3^loxP^	Cerebrum-specific depletion of HDAC3 causes abnormal neocortical lamination and hippocampal development, premature neurogenesis, disrupts neuronal migration, augmented DNA damage response and apoptosis, which contribute to depletion of neural stem and progenitor cells (NSPCs).	(77)

Abbreviations: ACTH, adrenocorticotropic hormone; ASO, antisense oligodeoxynucleotides; CeA, central nucleus of amygdala; CRH, corticotropin-releasing hormone; HDAC, histone deacetylase; NCOA, nuclear receptor coactivator; NCOR, nuclear receptor repressor; OVX, ovariectomized; POMC, proopiomelanocortin; T3, triiodothyronine; T4, thyroxine; TH, thyroid hormone; TSH, thyrotropin (thyroid-stimulating hormone); VMH, ventromedial hypothalamus

Different splicing variants of NCOA1 can have different roles in regulating glucocorticoid signaling. The NCOA1a variant is abundant in the PVN and can contribute to the repression of CRH gene expression. The NCOA1e variant is abundant in the CeA and seems to be lacking the repressor activity (54). Injection of “exon-skipping” antisense oligodeoxynucleotides targeting the 1e-specific exon in the CeA can induce a shift in the expression ratio of the 2 splice variants in favor of 1a, without adverse effects or activation of compensatory mechanisms (55). Such a shift of the NCOA1 splice variants blunts glucocorticoid-induced exploratory behavior in the open-field test and attenuated context-dependent fear memory, but does not change cue-dependent fear memory (56).

NCOA2^-/-^ mice display significantly lower basal corticosterone levels and a blunted corticosterone secretion in response to emotional and physical stress. However, this phenotype can be due to the NCOA2 function in adrenal gland rather than in the brain, because aberrations in the structure and function of the adrenal gland are observed in this mouse model (57). NCOA2^-/-^ female mice, but not males, show decreased anxiety and motor coordination. By comparison, NCOA2^-/-^ male mice show deficits in sensorimotor gating (50). NCOA3^-/-^ female mice show increased anxiety, whereas NCOA3^-/-^ male mice show normal explorative behaviors. Both male and female NCOA3^-/-^ mice show decreased nociceptive behaviors (50). Targeted metabolomics analyses identified reduced amino acid levels in NCOA3^-/-^ mice brain (13).

NCOR1^-/-^ and NCOR2^-/-^ mice are embryonic lethal, preventing the characterization of neuroendocrine or behavioral effects (58, 59). Injection of siRNA targeting NCOR1 into the amygdala of rats at 12 or 28 hours after birth reveals that NCOR1 knockdown increases juvenile social play behaviors in males and anxiety-like behaviors in both males and females. However, social interaction in the 3-chamber assay is normal in these rats (60). Tamoxifen-inducible postnatal global deletion of NCOR2 causes dramatic obesity in mice on a standard chow diet (61). The weight gain is associated with normal food intake, but a decrease in energy expenditure. It is unclear whether this phenotype is due to the NCOR2 function in the CNS.

### Hormonal signaling

NCOA1 plays an important role in the thyroid hormone (TH) signaling and central regulation of the hypothalamic-pituitary-thyroid (HPT) axis. Low circulating levels of TH, including triiodothyronine (T3) and thyroxine (T4), can trigger the hypothalamus to release thyrotropin-releasing hormone (TRH). TRH stimulates the anterior pituitary to produce thyrotropin (thyroid-stimulating hormone; TSH). TSH stimulates the thyroid to produce TH. High levels of circulating TH suppress the release of TRH from the hypothalamus and TSH from the anterior pituitary, which constitutes a negative feedback control (62). NCOA1^-/-^ mice display elevated circulating levels of TSH, T4, and T3 (63, 64). Suppression of TSH by the administration of supraphysiological doses of T3 results in a reduction of endogenous T4, suggesting that the high TH concentration in NCOA1^-/-^ mice is driven by TSH. Compared to WT control, NCOA1^-/-^ mice require higher doses of T3 to suppress TSH and T4, suggesting reduced sensitivity of the thyrotroph in the anterior pituitary to TH (63). These studies suggest that NCOA1, despite its name, is required for TH-mediated repression of TSH. This is in line with another study with TRβ. A mutation (E457A) in TRβ abolishes its interaction with NCOA but preserves normal T3 binding and NCOR interactions (6). Homozygous E457A mice display high circulating levels of T4, T3, and TSH, and high levels of *Tshb* gene expression in the pituitary, suggesting that T3-mediated suppression of TSH requires NCOA (65). The activator vs repressor function switch of NCOA1 is not entirely understood and might have something to do with interactions with different domains of TRβ (6). In summary, TRβ-NCOA1 interaction is required for both positive and negative regulation of gene transcription by TH.

Brain NCORs also play important roles in regulating the HPT axis. Cre/loxP-mediated deletion of the last few exons of NCOR1 leads to a truncated NCOR1 protein NCOR1ΔID (66). Expression of NCOR1ΔID postnatally results in lower circulating TH levels without a compensatory increase in TSH production, demonstrating that NCOR1 controls TH production and also the feedback loop through TSH. Pituitary-specific expression of NCOR1ΔID using a Cre driven by the glycoprotein α-subunit promoter (P-ΔID mice) shows similar low TH levels with decreased TSH production, suggesting that the phenomenon is due to a cell-autonomous function of NCOR1 in the pituitary. These results suggest that NCOR1, despite its name, is required for hypothyroidism-induced activation of TSH expression in the pituitary (67). This notion is consistent with another study with TRβ manipulation. The TRβ PV mutation was identified in a patient with resistance to thyroid hormone (RTH). The PV mutation abolishes the T3-binding activity and transcription capacity of TRβ in a dominant-negative manner (68). Mice expressing the TRβ PV mutant display RTH, including elevated TSH and TH levels, thyroid hyperplasia, and weight loss (68). These RTH phenotypes are partially rescued when mice are crossbred to the NCOR1ΔID mice, suggest a role of NCOR1 gain-of-function in PV-induced RTH. It is possible that the TRβ PV mutant is unable to effectively release NCOR1 and that NCOR1 contributes to the transactivation activity of unliganded TR.

### Learning and memory

Aside from the hormonal regulation, NCOA1 may also be involved in cognitive function such as learning and memory that may or may not involve hormones. NCOA1^-/-^ mice show reduced motor learning and nociception in males but not in females, compared with WT littermates (31, 50) (Table 1). The compromised learning in the global NCOA1^-/-^ mice may be due to developmental delay (31). In a separate mouse model, knockdown of NCOA1 in the adult mouse hippocampus through injection of the lentivirus expressing shRNA impairs spatial learning and memory in the Morris water maze test, decreases the expression of synaptic proteins and CA1 synapse density, and impairs *in vivo* long-term potentiation (69). In cultured hippocampal cells, synaptic protein levels are also decreased by NCOA1 knockdown, suggesting cell-autonomous regulation (69). These results suggest that NCOA1 has development-independent roles in learning and memory.

The depletion of NCOR1/2 in GABAergic neurons with Vgat-Cre (NS-V mice) causes learning and memory deficits (70). The electrophysiological analysis identifies the hyperexcitability of GABAergic neurons in the lateral hypothalamus (LH) in NS-V mice. Ribosome profiling identified that GABA_A_ receptor subunits are downregulated in the LH^GABA^ neurons in NS-V mice. This hypothalamic excitatory/inhibitory (E/I) imbalance impairs synaptic plasticity in the hippocampus CA3 region through a monosynaptic LH^GABA^-to-CA3 neural projection, which accounts for the neurocognitive dysfunction in NS-V mice (70).

NCOR1/2 is required for the enzyme activity of HDAC3. In a whole-body knock-in mouse model with mutations in the NCOR1/2 DADs that disrupt their binding to HDAC3 (NS-DADm mice), HDAC3 enzymatic activity is abolished in the brain and other tissues tested (70). These mice display social interaction deficits, impaired spatial learning and recognition memory, reduced anxiety, and enhanced locomotor coordination (Table 1**).** Gene expression profiling reveals altered expression of genes encoding GABA_A_ receptor subunits, along with several autism spectrum disorder (ASD)-related genes such as SH3 and multiple ankyrin repeat domains protein 3 (*Shank3)*, Forkhead box protein P2 (*Foxp2)*, and myocyte-specific enhancer factor 2c (*Mef2c)*. These results are consistent with the disrupted GABA signaling observed in the NS-V mice (70).

The effects of HDAC3 depletion on cognitive function are brain region–specific. HDAC3 depletion in adult mice in the hippocampal CA1 region through virus-mediated Cre recombinase enhances long-term memory formation (71). Depletion of HDAC3 in the nucleus accumbens using a similar approach enhances cocaine-induced conditioned place preference (CPP) acquisition, which is associated with increased gene expression during the consolidation phase of acquisition (72). Depletion of HDAC3 in the hippocampal CA3 through injection of AAV-Cre into the HDAC3 floxed mice does not affect learning or memory, while depletion of HDAC3 in the lateral hypothalamus impairs memory (70). Depletion of HDAC3 in retinal ganglion cells (RGC) in adult mice through viral approaches ameliorates RGC death, heterochromatin formation, nuclear envelope breakdown, and nuclear pore damage in RGCs following optic nerve crush (73).

In addition to the abovementioned roles in adult animals, HDAC3 also plays an essential role in brain development. Global HDAC3 deletion is embryonic-lethal (74). Conditional deletion of HDAC3 in mice using Nestin-Cre causes abnormal cytoarchitecture of the neocortex and cerebellum that leads to lethality within a day after birth (75). Mice depleted of HDAC3 in forebrain neurons as well as a few other brain regions using Thy1-Cre or calcium/calmodulin dependent protein kinase IIα (Camk2a-Cre) show the normal organization of cells within the cortex or of cerebellar Purkinje neurons at birth. However, these mice display progressive hindlimb paralysis, ataxia, higher numbers of astrocytes, and Purkinje neuron degeneration, which leads to lethality at approximately 6 weeks old (75). In another study, HDAC3 depletion with Camk2a-Cre causes neuron loss in the hippocampus, hyperactivity, abnormal exploratory behavior, hindlimb clasping, sociability deficits, and impaired memory (76). HDAC3 activates gene expression in the hippocampus through positively regulating FOXO activities (76). Cerebrum-specific depletion of HDAC3 by Emx1-Cre causes abnormal neocortical lamination and hippocampal development, premature neurogenesis, disrupts neuronal migration, augments DNA damage response and apoptosis, which all contributes to the depletion of neural stem and progenitor cells (NSPCs) (77).

## Genetic Variants in CNS-Related Conditions in Humans

While NCOA1-3 genetic variants are widely found in human cancer specimens (7), their involvement in the CNS has just started to emerge. Fifteen rare heterozygous missense variants in NCOA1 were recently found in individuals with severe, early-onset obesity using the exome sequencing or targeted sequencing data on 2548 obese individuals (42) (Table 2). Six out of 7 randomly selected variants show impaired interaction with STAT3 *in vitro* or compromised ability to mediate leptin-induced POMC gene transcription in a reporter gene assay in cultured cells, suggesting that the impaired leptin signaling could underpin the obesity phenotype in these individuals.

**Table 2.** NCOA and NCOR Complex Genetic Variants in Humans

Gene	Changes of Nucleotides or Amino Acid Sequence	Inherited or de novo	Phenotypes	Ref.
*NCOA1*	V136M, M381R, R385Q, N391S, Q463H, S557T, Q597P, S603C, A715T, S730R, S738L, T979P, M984T, P988S, P1034L, I1127T, N1212K, S1250I, L1376P	Inherited or unknown	Severe, early-onset obesity	(42)
*NCOR1*	c.2182 + 2T>G (splice donor site), c.2182 + 1G > T (splice donor site), P1025L, c.3449-1G>C (splice acceptor site)	de novo	Autism spectrum disorder, intelligence disability	(70, 78, 79)
*NCOR2*	R2296Q, S647L, E1328G and V467I	de novo	Autism spectrum disorder, intelligence disability	(70, 80, 81)
*HDAC3*	L266S	de novo	Learning difficulty	(70)
*TBL1XR1*	3q26.31q26.32 (175 507 453–177 095 072) del, 3q26.32 (176 025 379-177 377 006) del, 3q26.32 (176 221 801−176 929 584) del, G70D, Y446C, L282P, H441R, D370Y, D328G, P444R, H213Q, C325Y, Y446H, Y446S, I269YfsTer8, I397SfsX19	de novo or unknown	Autism spectrum disorder, developmental delay, intellectual disability	(83, 85, 86, 87, 88, 89, 90, 93, 94)
*TBL1X*	H453Y, Y458C, N365Y, A366T, W369R, and c.1312-1G>A (splice site), R339X	Inherited or de novo	Central hypothyroidism	(99, 100)

De novo genetic variants in NCOR1, NCOR2, and HDAC3 are found in pediatric patients with intellectual disabilities or ASD (Table 2). A heterozygous 152 kb deletion affecting the NCOR1 gene is found in a 10-year-old boy with ASD (70). Another 8-year-old boy with ASD harbors a de novo NCOR1 heterozygous variant at canonical splice donor site (c.2182 + 2T >G) (70). Interestingly, an adjacent variant (c.2182 + 1G >T), which affects the same splice site, was identified in a 3-year-old girl with ASD (78). Both mutations would cause exon skipping, reading frameshift, and production of a truncated NCOR1 protein or even reduced protein production from the affected allele. If a truncated protein is produced, it is predicted to lack the RD3 and all ID domains on the C-terminus. Another study identifies de novo missense [c.3122C > T (P1025L)] and splice-acceptor (c.3449-1G >C) NCOR1 variants from 2 ASD patients, respectively (79). In addition to NCOR1, 2 other pediatric patients with learning difficulties carry de novo variants in NCOR2 [c.6887G > A (R2296Q)] and HDAC3 [c.797T >C (L266S)] respectively (70). A heterozygous NCOR2 missense variant [c.1940C > T (S647L)] is found in atypical Rett syndrome patients (80). A patient with microcephaly and intelligence disability carries compound heterozygous variants [c.3983A > G (E1328G)] and [c.1399G >A (V467I)] of NCOR2 (81).

TBL1XR1 is critical for brain development, and its de novo mutations have been identified in neurological disorders (Table 2). Targeted sequencing of over 200 genes in more than 10 000 patients with ASD, intellectual disability, seizure, microcephaly, or macrocephaly identified 13 cases carrying disruptive mutations in TBL1XR1 (82). Targeted sequencing of more than 2000 ASD patients suggests that TBL1XR1 is 1 of the 6 genes with recurrent disruptive mutations that collectively contribute to 1% of sporadic ASD (83). Another study did not find TBL1XR1 among ASD risk genes (84). TBL1XR1 mutations were also identified in isolated ASD cases. For example, deletion of 1.6 Mb (175 507 453–177 095 072) in the 3q26.31q26.32 region, or a 1.3 Mb deletion in the 3q26.32(176 025 379-177 377 006) region that includes TBL1XR1 gene, causes intellectual deficiency (85, 86). A girl with a 708 Kb microdeletion on chromosome 3q26.32 (176 221 801−176 929 584), encompassing only TBL1XR1, has an intellectual disability and brain malformation (87). 309Kb and 521Kb microduplications of TBL1XR1 leads to developmental delay, intellectual disability, ASD, and hearing loss (88). A de novo missense TBL1XR1 mutation [c.209 G > A (G70D)] is identified in a Japanese girl with West syndrome and ASD features (89). Whole-exome sequencing of Pierpont syndrome patients identifies Y446C, C325Y, and Y446H variants in TBL1XR1 as potentially disease-causing (90-92). TBL1XR1 Y446S is found in autosomal dominant mental retardation (93). A de novo missense mutation (L282P) and a frameshift mutation (I397SfsX19) are found in patients with ASD (83). Six de novo mutations of TBL1XR1 (H441R, D370Y, D328G, P444R, H213Q, and I269YfsTer8) were identified in patients with global developmental delay in the Deciphering Developmental Disorders (DDD) study (94).

TBL1X deficiency was originally found in cases with sensorineural deafness related to ocular albinism (95). Genetic variants of TBL1X were recently found in isolated pediatric patients with central hypothyroidism (CCH), an underdiagnosed disorder characterized by low circulating thyroid hormones due to insufficient stimulation by TSH without primary defects in the thyroid gland (96). The disease derives from the abnormal function of the pituitary gland or the hypothalamus due to congenital defects or environmental factors. Genetic variants in TSH-releasing hormone receptor (TRHR) and TSH have been found in isolated CCH (97). Recently, de novo or inherited mutations in TBL1X were found in isolated CCH cases in pediatric patients (98). Six missense mutations in TBL1X were identified from 6 families with CCH individuals, including c.1510C-T (H453Y), c.1526A-G (Y458C), c.1246A-T (N365Y), c.1249G >A (A366T), c.1258T > C (W369R), and c.1312-1G > A (splice) (99). All affected individuals show low free T4 with normal or high TSH. Most affected individuals had mild to profound hearing loss. Another de novo mutation, c.1015C-T (R339X), was found in a boy who was diagnosed with CCH at 6 years of age (100) (Table 2). *In vitro* cell studies show that some of these mutations reduced TBL1X protein levels (99), presumably due to disrupted folding, protein stability, or nonsense-mediated RNA decay. Compared with wild-type TBL1X, N365Y or Y458C show impaired co-activation of TRH and TSHβ gene transcription by unliganded TR in the presence of NCOR1 in a hypothalamic neuronal cell line (101). These studies suggest that the loss-of-function on TBL1X gene might cause CCH.

## Conclusion

We are at the dawn of elucidating the function of NCOAs and NCORs in the CNS. Genetic variants of these coregulators and their stable binding partners have been identified in pediatric patients with CNS-related conditions. Humanized genetic animal models with knock-in mutations from these human patients will be helpful in establishing a causative role of these genetic variants in the etiology of these conditions. The function of coregulators in the brain is highly dependent on the brain region, cell type, or developmental stages. Future studies using animal models with temporally controlled genetic manipulations in a specific brain region, cell type, or a circuit-related neuron population would help clarify their functions. At the molecular level, given the inability to directly bind to DNA, it is pivotal to identify and characterize the nuclear receptors (NRs) or transcription factors (TFs) that sense or relay upstream signals in each scenario. The posttranslational modification and regulatory mechanism of the coregulators in the context of the CNS activity is another area that warrants further investigation. Finally, despite their names, both NCOAs and NCORs can function as transcription activators and repressors. The molecular mechanism underpinning such a functional switch is unclear. It may depend on the chromatin context, the relative abundance of the coregulators, nuclear receptors, ligands, as well as other epigenome modifiers, readers, remodelers, and the transcription machinery assembly dynamics. In summary, the coregulators integrate distinct hormonal or environmental cues through binding to many NRs or TFs in a signal-dependent manner. Coregulators function as the master genomic regulator through coordinating a variety of biological processes in the brain.

## Abbreviations

ACTH: adrenocorticotropic hormone
AD: transactivation domain
AR: androgen receptor
ARC: arcuate nucleus
ASD: autism spectrum disorder
CARM: coactivator-associated arginine methyltransferase
CeA: central amygdala
CNS: central nervous system
CRH: corticotropin-releasing hormone
DAD: deacetylase activating domain
ER: estrogen receptor
GR: glucocorticoid receptor
HDAC: histone deacetylase
HLH-PAS: helix–loop–helix-PER-ARNT-SIM
HPT: hypothalamic-pituitary-thyroid
ID: interaction domain
NCOA: nuclear receptor coactivator
NCOR: nuclear receptor repressor
NR: nuclear receptor
NRID: nuclear receptor interaction domain
OVX: ovariectomized
POMC: proopiomelanocortin
PVN: paraventricular nucleus
RD: repression domain
shRNA: small hairpin RNA
S/T: serine/threonine-rich
T3: triiodothyronine
T4: thyroxine
TBL1X: transducin β-like 1 X-linked
TBL1XR1: transducin β-like 1 X-linked receptor 1
TH: thyroid hormone
TRH: thyrotropin-releasing hormone
TSH: thyrotropin (thyroid-stimulating hormone)
VMH: ventromedial hypothalamus
WT: wild-type

## Additional Information

***Disclosure Summary:*** The authors declare no actual or potential competing financial interests.

***Data Availability:*** All data generated or analyzed during this study are included in this published article or in the data repositories listed in References.
